# The buddy group – peer support for the bereaved

**DOI:** 10.1080/17571472.2018.1455021

**Published:** 2018-03-28

**Authors:** Susan Gerry Riley, Katherine Irene Pettus, Julian Abel

**Affiliations:** a Weston Hospicecare, Weston-super-Mare, UK; b International Association for Hospice and Palliative Care, Iahpc, USA; c Vienna NGO Committee on Drugs, Iahpc, USA; d Compassionate Communities, Helston, UK

**Keywords:** Bereavement, community development, public health

## Abstract

We describe in this paper the story of the ‘Buddy Groups’ for bereaved people that were set up at Weston Hospicecare in 2008 and have endured ever since. The group have helped bereaved people to find meaning and value despite their grief. We observed that, through the strength of the relationships formed, people were able to recover well. Group members reported back to us the significant value they placed on being in a Buddy Group.

## Why this matters to us

As palliative care clinicians, we are aware of the severe effect that bereavement can have and the lack of bereavement services to help those in need of support. We realised that significant numbers of people suffered in silence but at the same time did not require specialist bereavement care. We therefore developed the Buddy Group that was simple to set up, did not require prolonged professional input and was open to all who could benefit. We believe that this model could be used in a widespread way to support those in need.

## Key message

The morbidity and mortality that accompanies bereavement can be severe and includes suicide, death, depression, increased illness with many poor outcomes. The majority of bereaved friends and relatives suffer in silence, without support. When professional support is present, this is mostly in the form of one to one therapy. We describe a peer support group approach to bereavement which has the potential to be applicable broadly to the population and at low cost. The outcomes from the group are positive and will hopefully provide a springboard for further developments.

## Introduction

The Weston HospiceCare Buddy Group is a peer support group for people who have been bereaved (when someone they were close to had died). It arose from Weston HospiceCare courses for patients and their carers (Sharing and Travelling the Journey) about how to manage a terminal illnesses.

Recently bereaved people who have been in a long relationship or a caring role for several years may struggle to socialise as a single person in their community [1]. The loss of someone close can be a challenging and painful experience [2,3]. When someone has been a long term caregiver, the bereavement journey can be arduous [4,5]. Loss of meaning, value and identity are well recognised problems of bereavement, with social isolation and reduced social networks. The consequences of this can be severe, with increased mortality, physical morbidity, depression and suicide [6–8]. Re-establishing social networks is difficult after what can be many decades of partnerships. For example, women in particular do not like going to the theatre or coach trips alone when recently bereaved.

Bereavement is, however, a normal process that can be managed with supportive networks, normalising the experience [9,10]. Professional care can then be targeted at those with complicated grief reactions. Peer support networks make it possible to provide bereavement care, without seeing bereavement as a pathology that needs treating.

## Development of the buddy group

The Weston HospiceCare pre-bereavement courses (Sharing and Travelling the Journey), were set up in 2006 to help patients and carers manage a terminal illness. A number of bereaved carers, who had previously been attending these courses, wanted to continue to meet on a regular basis to share their experiences of grief. This led to the formation of the Buddy Group in 2008.

Initially the Buddy Group met monthly at the hospice, allowing participants to form strong relationships. Members gradually became confident and planned social occasions such as trips to cinema, lunches, day trips and weekly coffee mornings. At the meetings they were able to express their emotions and develop further their friendships. The hope and aim was that this group would encourage bereaved people to find a positive way forward with peer support and friendship.

Other recently bereaved people were invited to join. This became the pilot group (Buddy Group 1) and each year more groups were established. The members chose the name ‘The Buddy Group’. The term friendship or bereavement did not feel appropriate for them. The term ‘Buddy’ remained for all future groups. To date 150 people since September 2008 have taken part in a Buddy Group. We are now on Buddy Group 6 and about to launch Buddy Group 7.

The experience of the groups so far is that long lasting friendships are formed. As the groups moved forward, two previous carers assumed the role of facilitating the groups and arranging the events. We saw the benefit of on-going relationships and became keen for the groups to continue. At the same time, we did not want to build dependency on the hospice. We were keen for the groups to be self-sustaining. This is in keeping with the principles of participatory development which seeks to engage people in development projects. Participatory development has taken a variety of forms since it emerged in the 1970s as an important part of the ‘basic needs approach’ to development [11]. One underpinning principle of the approach is that better solution to problems are found, less by professional advice and more by those undergoing the experiences. The hospice had a role in facilitating this process rather than leading it.

The Hospice is still used as a place for the Buddy Group to meet. Support from previous caregivers continues to be offered as volunteers who facilitate the group. Meetings happen each month for two hours. It has no structured therapy sessions, unlike the pre-bereavement courses.

## Feedback

Box 1 Describes selected quotes from the Buddy Groups from feedback that was routinely provided (focus groups) to allow learning from each stage to inform the next.

Box 1. Buddy Group feedback

We are all at different stages of grief and understand what we each go through. It is normal to grieve. I don’t need counselling, just empathy and sympathy from someone who has been through a caring role and lost a spouse through death.

I am able to talk about my spouse. It is hard to go out with friends who are couples. In the Bubby Group we are new friends, sharing grief, memories and we do not feel so isolated.

Going out with family and friends without my wife was difficult. They didn’t want me to talk about her as they felt uncomfortable. I liked to talk about her with happy memories too. I can do this in the Buddy Group.

During those 6 ½ years there have been many challenges that have come our way. Without our loved ones those challenges can be really, really hard to face. We have faced this together and supported each other. We have had more cancer, a member having stroke and lots of illnesses.

For me personally, soon after my husband died both my parents died suddenly from heart attacks. These guys were there for me. They made sure – have you eaten dinner, have you eaten properly all this kind of thing. They said we’re here for you, we’re here for you. I can’t describe I have so much passion for all these people. They are my friends. They are like an extended family.

I have made a number of life- long friends. I have enjoyed holidays here in the UK and elsewhere. Huge trips that I wouldn’t have otherwise done. It aided the bereavement process and a big change in my life.

Box 2 Displays our learning from participant feedback and our own observations.

Box 2. Learning from Buddy Group feedback

Attrition rates are a normal part of groups as people choose to leave, not feeling the need for ongoing support

Groups are best kept to a maximum of 16 members at a time

Members of different Buddy Groups need to remain separate, since they are at different stages

Men often declined to attend when there were no other men in the group

People of working age were unable to attend weekday meetings

Some find it difficult to get to meetings and other buddies are often prepared to offer lifts when they have established relationships

Recently bereaved people may need some time alone before they are ready to join a Buddy Group

## Further community development initiatives

The feedback that ‘men declined to attend unless there were other men in the group’ resonated with previous work that recognised that men do not benefit in the same way as women [12–14]. This led the hospice to pilot a ‘Buddies in Shed’ group in 2015. This was aimed at bereaved men who would like social interaction in a ‘shed’ environment on a weekday and also on a Saturday to reach those also employed. Volunteers and previous bereaved men organised and took ownership of the scheme. We started a walking group for people who have been bereaved in 2016 that many found useful.

## Discussion

The model of the Buddy Group, especially when arising from pre-bereavement courses, is likely to be replicable in other places. The principle of the groups being self-supporting and self-sustaining means that they could continue as long as the participants feel it is valuable and necessary. Host organisations, in this case Western HospiceCare, need to be flexible in developing such groups, allowing the groups to take control of running the groups when they are ready [15].

The initial development of a Buddy Group may need facilitated support within a safe environment. The bereavement journey is supported by fellow group members which results in members overcoming fear of developing new social networks, preventing isolation and loneliness. Facilitation is concerned with building friendship rather than professional support.

The Buddy Group and the associated pre-bereavement courses can be thought of as a social movement that normalises the process of death and bereavement and turns it into a positive moment of transformation for those involved. We need more of this in the NHS.

## Disclosure statement

No potential conflict of interest was reported by the authors.
